# Intraoperative Assessment of Cochlear Nerve Function During Cochlear Implantation Using the Auditory Nerve Test Stimulator

**DOI:** 10.3390/audiolres15020036

**Published:** 2025-04-01

**Authors:** Karin Hallin, Nadine Schart-Morén

**Affiliations:** Department of Surgical Sciences, Otorhinolaryngology and Head and Neck Surgery, Uppsala University, 75185 Uppsala, Sweden

**Keywords:** cochlear implant, electrical auditory brainstem responses, auditory nerve test stimulator, long-term deafness

## Abstract

Background/Objectives: A crucial factor for a successful cochlear implant (CI) outcome is an intact auditory nerve (AN). The integrity of the AN can be tested during implantation by measuring electrical auditory brainstem responses (eABR) via the CI. A method that does not require a CI is the use of the auditory nerve test stimulator (ANTS) from MED-EL (Innsbruck, Austria). The aim of the current study was to investigate the cases tested with the ANTS at our clinic and to describe the hearing results following CI for the cases who were implanted with a CI. Methods: All patients underwent preoperative magnetic resonance imaging (MRI) and high-resolution computed tomography (HRCT) to rule out cochlear malformation or retrocochlear pathology. In this study, we described all cases from when we began using the ANTS in 2011. Results: Five patients were tested intraoperatively: three adults with long-term deafness prior to CI and two children with no detectable AN. Three of the five patients were implanted with a CI. All implanted patients in this study could hear with their CIs, even though the speech perception results were limited. Conclusions: The ANTS can be used as a method to assess cochlear nerve function during implantation. The eABR results from the ANTS and the implanted CI were comparable for all cases in our study. Minor changes in waveform latencies were found between ANTS and CI stimulation and may be explained by the insertion depth of the electrode used for stimulation.

## 1. Introduction

Cochlear implantation (CI) is a successful treatment for severe sensorineural hearing loss (SNHL). Many factors contribute to the hearing outcome following CI: cause of SNHL, age at implantation, presurgery speech perception, pre- or postlingual SNHL, deafness duration, etc.

A crucial factor for achieving a successful CI outcome is an intact auditory nerve (AN). The integrity of the AN can be tested during implantation by measuring electrical auditory brainstem responses (eABRs) using the CI as the stimulator and an evoked potential (EP) system as the recorder. This method is well described and is a well-recognized method for AN assessment [1]. However, the method demands that a CI be implanted. A method that does not require a CI is the auditory nerve test stimulator (ANTS) from MED-EL (Innsbruck, Austria). The ANTS is a simplified electrode array that can be used to measure eABR prior to CI. In cases lacking positive eABR results, the surgery can be terminated without implantation of the CI. Lassaletta et al. [2] used ANTS as a tool to decide whether to place a CI in patients with vestibular schwannomas (VS). Cinar et al. [3] described the role of the ANTS in decision-making between CI and auditory brainstem implant (ABI).

It has been debated whether there is a time limit for deafness duration in determining a CI candidate [4]. An earlier study at our clinic showed that even after nearly 50 years of deafness, it is possible to gain monosyllable speech perception [5]. However, several studies have shown that the duration of deafness seems to negatively affect speech perception [6,7]. In addition, the age at onset of deafness affects CI speech perception in cases with long-term deafness [5,8].

At our clinic, the ANTS has been used to test the integrity of the AN before CI in a few cases. The aim of the current study was to investigate our cases tested with the ANTS and describe the hearing results following CI for the patients who were implanted with a CI. The study was approved by the Swedish Ethical Review Authority (Dnr 2014/437, 19 November 2014) and written informed consent for the study was given by the patients implanted with a CI; thus, their results are described in detail in this study.

## 2. Materials and Methods

### 2.1. Patients

All patients were referred to our clinic (tertiary CI center) for cochlear implantation or auditory brainstem implantation. They underwent preoperative magnetic resonance imaging (MRI) and high-resolution computed tomography (HRCT) to rule out cochlear malformation or retrocochlear pathology. Cochlear implantation was performed via a transmastoid posterior tympanotomy approach under general anesthesia. The ANTS as well as the CI were inserted via the round window. In this study, we reviewed the data of the five cases that were examined at our clinic since we started using the ANTS in 2011. The indication for using ANTS in our clinic is a doubtful prognosis for CI rehabilitation in cases of uncertain cochlear nerve function. The ANTS electrode array was implanted by different surgeons for the patients in this study. The eABR response was interpreted by the same engineer for all patients.

### 2.2. The ANTS

**Figure 1:**
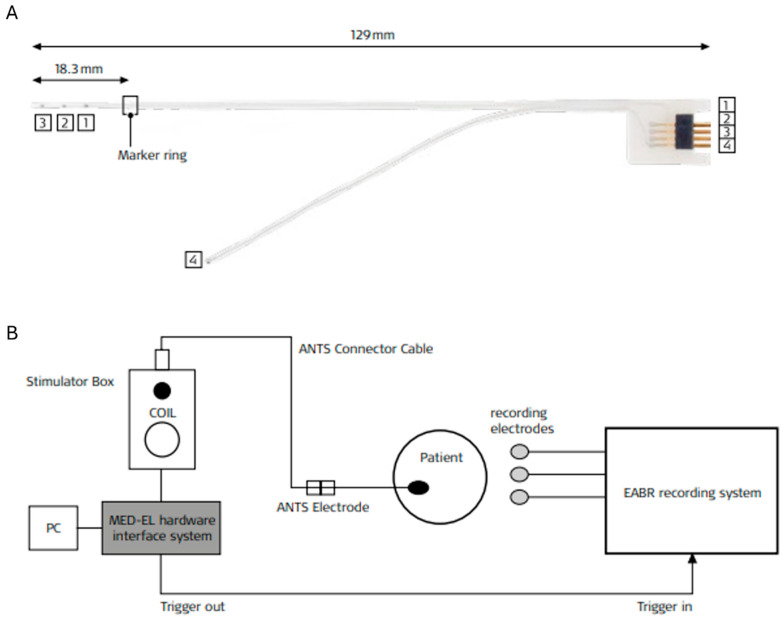
(**A**) The ANTS electrode array with electrode contacts 1–4. The apical electrode (3), medial electrode (2), basal electrode (1), and reference electrode (4). (**B**) The stimulation and eABR recording setup. PC—computer with MedEl programming software (©MED-EL, Innsbruck, Austria).

The ANTS is an 18 mm long electrode with three contacts (nos. 1 to 3) and an extracochlear reference electrode (no. 4) (Figure 1A). CI programming software from MED-EL (versions 7.0.2 to 10.0.2) is needed for stimulation. The MAX Hardware Interface System and the ABI Stimulator Box from MED-EL are also needed. Bipolar stimulation on the ANTS can be carried out between any two of the four electrode contacts at various pulse durations and amplitudes. The ANTS and the stimulation/recording setup have been described in detail in Medina et al.’s study [9]. For stimulations in this study, amplitude varied from 100 to 1000 cu. A 1 cu is equal to 1 μA, and this is a unit used by MED-EL for CI programming. The pulse duration was either 53 or 60 μs. The unit used for setting the most comfortable levels (MCLs) in the sound processor in the MED-EL programming software is qu and equals pulse width times amplitude (electrical charge in nC).

### 2.3. Recording with the EP System

To record the eABR from the ANTS stimulation, needle electrodes were placed on the vertex (positive), contralateral mastoid (negative), and forehead (ground). The needle electrodes were connected to an EP system to record the eABRs. The EP system was triggered to record from the MED-EL MAX hardware interface system. The EP system Otometrics Chartr 200 (GN Otometrics, Taastrup, Denmark) was used for the eABR recordings. The EP system averaged 1000 sweeps and was filtered by a low-pass filter at 5 kHz and a high-pass filter at 5 Hz. A detailed description of how eABRs are measured at our clinic as well as their prognostic value on hearing outcome is found in Lundin et al. [10]. A clear detectable wave V in the eABR response was considered a positive response in the current study. The stimulation and eABR recording setup are displayed in Figure 1B.

### 2.4. Speech Perception

Speech perception with CI was measured by a Swedish three-digit test and bisyllabic word (BS-word) and monosyllabic word (MS-word) tests. The speech perception measures were conducted in a sound-treated booth in a free field at a level chosen by the patient for the three-digit and BS-word tests and at 65 dB sound pressure level (SPL) for the MS-word test. The tests were conducted with only the implanted ear with a loudspeaker in front of the patient. No presurgery speech perception tests were performed, as all patients in this study were deaf in the ear to be implanted and had no detectable hearing thresholds.

## 3. Results

Five patients were tested intraoperatively with the ANTS at our clinic. There were three adults with long-term deafness prior to CI (50–60 years), and two children had no detectable AN from an MRI. The patients are summarized in Table 1.

The eABR recordings via the ANTS and from the implant are described below for the patients with a positive response. The hearing outcome from the CI is also described for each patient. Patients 1 and 2 were both children with no presurgery detectable hearing, as well as no detectable AN from MRI. They both showed no eABRs when tested using the ANTS. Patient 1 was implanted with an ABI. For patient 2, it was decided to use the ANTS during implantation and to only implant the CI if there were positive results from eABR.

### 3.1. Patient 3

**Figure 2:**
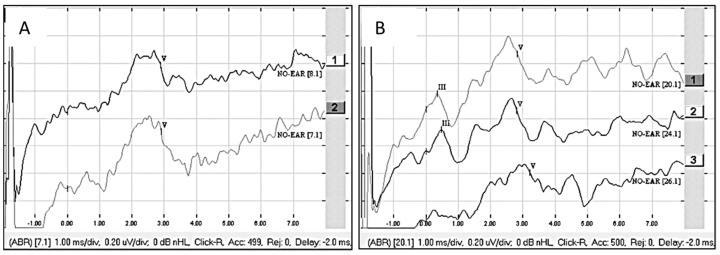
(**A**) eABR from ANTS, stimulation on electrode contacts 3 and 4 at 30 qu [1] and 42 qu [2]. (**B**) eABR from CI, stimulation at 30 qu on electrode contacts 1 [1], 7 [2], and 11 [3]. Observe that there is a 2 ms delay on the stimulus window when studying the waveform latencies.

Patient 3 was presumably deaf in the right ear from the age of 8, but this was not certain as no audiograms were available before the age of 8. The deafness duration was at least 55 years in the ear to be implanted. There was hearing loss in the left ear for 35 years, and the patient was using a hearing aid in that ear. The ANTS was stimulated on electrode contacts 3 and 4 with a pulse width of 60 μs at 100 cu (6 qu), 500 cu (30 qu), and 700 cu (42 qu). A clear wave V was detected at the 500 cu (30 qu) stimulation and raised in amplitude at the 700 cu (42 qu) stimulation (Figure 2A). Additional stimulations on electrode contacts 1 to 3 as well as 1 to 4 also gave a clear eABR wave V at 500 cu (30 qu) stimulation. The patient was implanted with a Synchrony 2 Flex 28 (MED-EL) electrode, and eABR was measured when stimulating electrodes 1, 7, and 11 at a pulse width 30 μs and an amplitude of 1000 cu (30 qu). Clear waves III and V could be seen from stimulation on electrodes 1 and 7 and V from stimulation on electrode 11 (Figure 2B). At first fitting, the patient was unsure if the stimulation gave hearing or some other sensation in the head. However, after one week, it became clear that implant stimulation gave hearing sensations. The patient had been recently fitted. One month after fitting, the patient scored 5% on the three-digit test and used the implant 3 h per day. Three months after fitting, the patient had increased usage to 5 h per day. Speech tests showed no additional improvement at three months, but the patient believed that the CI hearing was progressing and gave access to environmental sounds and to some extent directional hearing.

### 3.2. Patient 4

**Figure 3:**
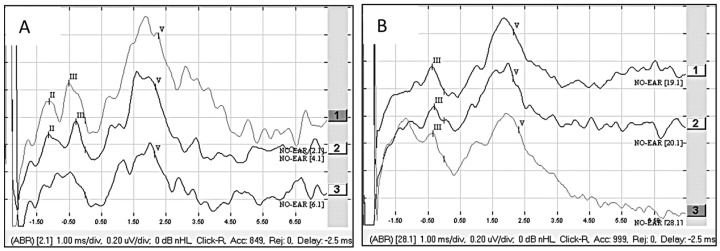
(**A**) eABR from ANTS, stimulation on electrode contacts 3 to 4 at 31.8 qu [1], 39.75 qu [2], and 53 qu [3]. (**B**) eABR from CI, stimulation at 30 qu on electrode contacts 1 [1], 7 [2], and 11 [3]. Observe that there is a 2.5 ms delay on the stimulus window when studying the waveform latencies.

Patient 4 was presumably deaf in the left ear from the age of 5. However, this was not certain as no audiograms were available before the age of 5. The deafness duration was at least 60 years in the ear to be implanted. The patient had hearing loss for 47 years in the right ear and used a hearing aid in that ear. The ANTS was stimulated on electrode contacts 3 to 4 with a pulse width of 53 μs at 600 cu (31.8 qu), 750 cu (39.75 qu), and 1000 cu (53 qu). A clear wave V was detected at the 600 cu (31.8 qu) stimulation and with raised amplitude at the 750 cu (39.75 qu) and 1000 cu (53 qu) stimulations (Figure 3A). Also, waves II and III could be detected from stimulation at 600 cu (31.8 qu) and 750 cu (39.75 qu). Additional stimulations on electrode contacts 2 to 4 as well as 1 to 4 also gave a clear eABR wave II, III, and V at 750 cu (39.75 qu) stimulation. The patient was implanted with a Synchrony Flex 28 electrode, and eABR was measured when stimulating electrodes 1, 7, and 11 at a pulse width 30 μs and an amplitude of 1000 cu (30 qu). Clear waves III and V could be seen from stimulation on electrodes 1, 7, and 11 (Figure 3B). At first fitting, the patient could not tell if the stimulation was soft or strong but obtained hearing sensations from CI stimulation. After one week, the patient heard the CI stimulation as a modulated noise. After one month, the patient scored 8% on the three-digit test, used the CI full-time (>8 h/day), and practiced the CI hearing by listening to audio books and music. The tinnitus in the implanted ear decreased after implantation. After one year, the patient scored 29% on BS-words and 8% on MS-words. Currently, the patient has had the CI for eight years, and the speech perception is still at the same level as it was after one year. However, despite the modest speech perception, the patient is very satisfied with the CI and uses it full-time. The patient is grateful for all the environmental sounds that are heard via the CI and enjoys listening to music with the CI.

### 3.3. Patient 5

**Figure 4:**
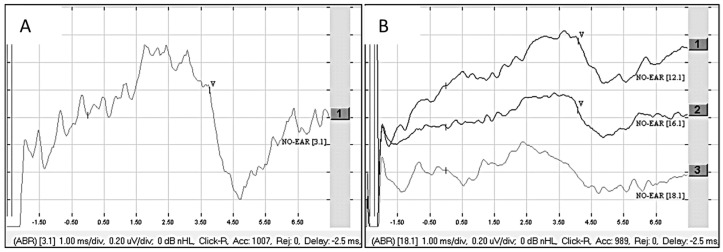
(**A**) eABR from ANTS, stimulation on electrode contacts 3 and 4 at 45 qu [1] and 42 qu [2]. (**B**) eABR from CI, stimulation at 39 qu on electrode contacts 1 [1], 7 [2], and 11 [3]. Observe that there is a 2.5 ms delay on the stimulus window when studying the waveform latencies.

The patient was presumably deaf in the left ear from the age of 6, but it was possible that they had been deaf from an earlier age. The deafness duration was at least 50 years in the ear to be implanted. There was hearing loss for 16 years in the right ear, and a hearing aid was used in that right ear. The ANTS was stimulated on electrode contacts 3 to 4 with a pulse width of 60 μs at 750 cu (45 qu), and a clear wave V was detected (Figure 4A). Additional stimulations on electrode contacts 2 to 4 at 750 cu (45 qu) gave a clear wave V with raised amplitude when stimulated at 850 cu (51 qu). The patient was implanted with a Synchrony Flex Soft electrode, and an eABR was measured when stimulating electrodes 1, 7, and 11 at a pulse width 30 μs and an amplitude of 1000 cu (30 qu). No clear wave V could be found from that stimulation. Raising the stimulation to pulse width 60 μs and an amplitude of 650 cu (39 qu) gave a clear wave V when stimulating electrodes 1 and 7 (Figure 4B). At first fitting, the patient obtained hearing sensations from CI stimulation as well as some facial nerve stimulation. The sound was perceived as noise, and the patient perceived all sounds coming from the right ear (the not implanted ear). After one week, the CI was still noisy and unclear, and the patient got tinnitus from using the CI. They could hear some environmental sounds. There was no speech perception with the CI after one month, and the CI disturbed the hearing aid, making the bilateral hearing worse when using the CI. The patient used the CI full-time but took it off to hear better in some situations. There was still no speech perception with the CI after one year, and audiological testing made it clear that the CI made the bilateral hearing worse. After two years, the patient scored 65% on the three-digit test, and the CI no longer disturbed the bilateral hearing. At that point, the patient used the implant 2 h per day. After five years, the patient scored 80% on the three-digit test and 12% on the BS-word test. The bilateral MS-word test showed better results using the CI and the hearing aid together compared with using the hearing aid only. The speech perception with the CI has not improved since the 5-year appointment, and the patient uses the CI approximately 1 h per day. Today, the patient is also implanted in the right ear and uses that CI full-time and the left implant a few hours a day.

## 4. Discussion

The ANTS can be used as a method to assess cochlear nerve function during implantation. The eABR results from the ANTS and the implanted CI were comparable for all cases in our study, with positive outcomes from eABR with the ANTS. Minor changes in waveform latencies were found between ANTS and CI stimulation and can be explained by the insertion depth of the electrode used for stimulation. The eABR waveform latencies increase toward cochlear base stimulation [10].

All implanted patients in this study could hear with their CIs, even though the speech perception results were limited. In conjunction with the findings by Cinar et al. [3], patients with no detectable wave V from eABR using the ANTS and no detectable AN following HRCT and MR were not implanted with a CI. Vesseur et al. [11] suggested that a negative eABR can be used as an indicator for ABI. However, the consensus statement from the Third International Pediatric ABI Meeting [12] warned that there is the possibility of false negative results, that is, that the test can be negative in the presence of a hypoplastic CN due to anatomical abnormalities in the cochlea.

Patients 3 and 4 showed a clear eABR wave V from both ANTS and CI stimulation. From CI stimulation, wave III was also present. Even though the waveform latencies were found to be higher than average, the average CI eABR wave V latency for the low-frequency region in the cochlea being 3.99 ms [10], the waveform structure was normal. Also, the waveform latency was not used as a factor to decide a positive outcome in this study. Patient 3 has had the implant for three months only and it is too early to evaluate the CI speech perception result. Patient 4 had below-average results from speech perception tests, scoring 8% on the MS-word test (the average score at our clinic was 39.5% [13]). Also, Patient 4 is a full-time user and is very satisfied with the CI hearing.

For Patient 5, it took two years of full-time usage before the CI stopped disturbing the contralateral hearing and making the bilateral hearing worse, and it took five years for the CI to contribute to the bilateral hearing. That long rehabilitation time before gaining a positive outcome from the CI might have caused many patients give up and to regret the decision to implant a CI. Moreover, analyzing the eABRs from the ANTS and the CI showed an unusually late wave 5 latency (>6 ms) at an unusually high stimulation level (>40 qu). In Cinar et al.’s study [3], testing eABR on 11 subjects with ANTS, no wave V latencies were seen >6 ms. Also, the waveform structure was not optimal and not considered normal, even though there were no questions about a clear detectable wave V. Perhaps that should have led to the decision not to implant a CI in that case.

## 5. Conclusions

Our study results indicate that eABR using the ANTS can be used as a reliable method for testing AN integrity before CI. However, there is a need for further investigation into stimulation levels as well as eABR waveform latencies and waveform quality and how those factors predict CI hearing outcomes.

## Figures and Tables

**Table 1:** Age at surgery (years), reason for using the ANTS electrode, detectable wave V from ANTS-stimulation (yes/no), implant that the patient received (CI/ABI and model), detectable wave V from implant-stimulation (yes/no), implanted ear (left/right).

Patient No.	Age at Surgery	Reason for ANTS	Detectable Wave V from ANTS Stimulation	Implant Type:	Detectable Wave V from Implant Stimulation	CI Implanted Ear
1	2	No detectable auditory nerve	No	ABI: Cochlear ABI 541	Yes	
2	3	No detectable auditory nerve	No	Not implanted	Not implanted	
3	63	Deafness duration ≈ 55 years	Yes	CI: MED-EL Synchrony 2 Flex 28	Yes	Right
4	65	Deafness duration ≈ 60 years	Yes	CI: MED-EL Synchrony Flex 28	Yes	Left
5	56	Deafness duration ≈ 50 years	Yes	CI: MED-EL Synchrony Flex Soft	Yes	Left

## Data Availability

The data presented in this study are available on request from the corresponding author due to ethical restrictions.
